# Medicine as a Business and Business in Medicine

**Published:** 1868-06

**Authors:** 


### Editorial Department.

### Medicine as a Business and Business in Medicine.

The practice of medicine as a business, in no way favorably commends itself to the attention of those about to choose a profession. It demands exclusive, unremitting and earnest attention, and with all this, offers but the very poorest return. It does, it is true, afford a few who are faithful and active enough to gain distinction, a fair income, but the great mass of hard workers receive "small wages." Attention is not enough directed to the causes of this poor return for medical services. It has been common for physicians themselves to place little pecuniary value upon their services, and to demand little or nothiug from the community. There are few cities or towns in which cannot be found men who are called *because* they never *demand* any pecuniary return, perhaps indifferently guess at the amount due them if by chance some active business man values their services enough to insist upon payment. The great fault in medicine, as a business, arises mainly from the fact that so many who practice it, are not "business men;" have no rational idea of exchange. We have never known a physician habitualty selling himself for nothing, but received, really, *all* he was worth. The men who value their time and advice are almost always appreciated. Business men respect a consistant, straight-forward, reliable, prompt, business man; indeed, everybody appreciates such men. A careless, indifferent, unsystematic man, who never has system in his pecuniary matters, is the poorest, and as a rule, most unscientific, careless, unprogressive and unsatisfactory medical adviser in the world. It is all right that they expect nothing for their services; they are really of no value; a poor business man is a poor doctor.

Bnt we were about to suggest the importance to the profession and the advantages to the community of *early* and prompt presentation and settlement of medical bills. There is no other man who devotes his personal time to any pursuit or calling, but expects and demands his reward. There is nothing in the conditions of physicians' necessities and wants which justify delay in obtaining the rewards of their services. If by chance any are able to neglect collections, it constitutes no reason for so doing, and is a disadvantage to both physicians and the public. The time has fully come when medical men should expect immediate return, and when the public should understand that the physician is to be paid immediately, before anybody else—that his is a debt of honor, that bankruptcy does not effect the obligation, that the grocer and the dry goods merchant may be put off a little, but a physician who attends them at all seasons and hours, adds his sympathies and personal interests to theirs, bears the anxieties inseparable from his calling and faithfully advises them in times of pain and peril is to be rewarded. The public can never appreciate these facts and conditions until instructed to do it, and will undoubtedly be slow to learn, but the public can learn it, would have known it long before this, if the "slip-shod" business manners of physicians had not been constantly inculcating the opposite doctrine of delay. Who in the profession are best and soonest paid? Certainly those who are highest prized and most extensively patronized. The real business men of the profession charge for their services and collect their bills, and men say, "here is your claim; thank you, sir, for your kindness and attention; I feel thankful that I could obtain your services." The "slip-shod" physician receives pay after the following manner, by the hand of a child: "Dr. Waitforever: Dear sir: I am no better, and feel anxious about myself. I hoped, when I called you, that my sickness would be trifling, and that I might avoid expense, but as I do not improve, I desire to call Dr. ———, who is, as you say, very high in his charges and expects his pay down, but who is distinguished in the care of such cases. Yours, truly, True E. Conomy."

"The poor ye have with you always, and may do them good when ye will." It is wrong, and often cruel, to accept pay of the very poor when they are sick. If physicians will cultivate system and promptness in their business, they can advise and help the poor and be respected, and paid, or thanked, by all.

